# Cryopreservation of equine mesenchymal stem cells in 95 % autologous serum and 5 % DMSO does not alter post-thaw growth or morphology in vitro compared to fetal bovine serum or allogeneic serum at 20 or 95 % and DMSO at 10 or 5 %

**DOI:** 10.1186/s13287-015-0230-y

**Published:** 2015-11-26

**Authors:** Alexis Mitchell, Kristen A. Rivas, Roger Smith, Ashlee E. Watts

**Affiliations:** Department of Large Animal Clinical Sciences, Texas A&M University, College Station, TX 77843 USA; Department of Veterinary Pathobiology, Texas A&M University, College Station, TX 77843 USA

**Keywords:** Mesenchymal stem cell, Cryopreservation, Fetal bovine serum, Serum, Equine

## Abstract

**Introduction:**

Equine superficial digital flexor tendon injury is a well-accepted model of human tendon injury and is routinely treated with local injections of autologous mesenchymal stem cells (MSCs). Identification of a clinically safe medium for short-term cryopreservation of MSCs prior to cell implantation would streamline laboratory and clinical procedures for autologous regenerative therapies. Veterinary experience with short-term (MSCs prepared after the injury has occurred) cryopreserved MSCs in naturally occurring injury in the horse will be of value to human practitioners.

**Methods:**

Equine bone marrow derived MSCs were cryopreserved in 6 different solutions consisting of 20 % serum, 10 % DMSO and 70 % media or 95 % serum and 5 % DMSO. Serum was autologous serum, commercially available pooled equine serum or fetal bovine serum (FBS). Cell survival, morphology and growth kinetics were assessed by total cell number, measurement of growth kinetics, colony-forming-unit-assay and morphology of MSCs after monolayer culture post-thaw.

**Results:**

There were no significant differences in post-thaw viability, total cell number, morphology scores or growth kinetics among the 6 solutions. Post thaw viabilities from each group ranged from 80-90 %. In all solutions, there were significantly fewer MSCs and the majority (99 %) of MSCs remained in the original generation 24 hours post-thaw. Seventy two hours post-thaw, the majority of MSCs (50 %) were proliferating in the fourth generation. Mean colony count in the CFU-F assay ranged from 72 to 115 colonies.

**Conclusions:**

Each of the serum sources could be used for short-term cryopreservation of equine bone marrow derived MSCs. Prior to clinical use, clinicians may prefer autologous serum and a lower concentration of DMSO.

**Electronic supplementary material:**

The online version of this article (doi:10.1186/s13287-015-0230-y) contains supplementary material, which is available to authorized users.

## Introduction

The equine athlete is a well-accepted model for stem cell therapies in musculoskeletal injury [1]. This is because the horse suffers from naturally occurring superficial digital flexor tendon injury that is similar to humans, and culture-derived and expanded mesenchymal stem cells (MSCs) are being used to treat these injuries [2]. Use of clinical practices in equine cellular therapies that are acceptable in human medicine would be beneficial to help ascertain the value of stem cell therapy for tendon injury in this naturally occurring large animal model.

The ideal stem cell preparation, whether frozen or fresh, is an ongoing debate in medicine [3–6]. Cryopreserved MSCs are used in approximately 35 % of published MSC clinical trials [7]. However, in veterinary medicine the majority of laboratories preparing MSCs for horses throughout the world do so with fresh cells [8]. This is not to state that MSCs have not been previously frozen, but that immediately prior to implantation in the patient the MSCs are in monolayer culture and are prepared for injection immediately prior to clinical use with transport to the animal site in cooled media. Identification of a cryopreservation medium that allows for immediate clinical use of MSCs post thaw would be beneficial to streamline laboratory and clinical procedures and reduce associated costs. It is also possible that the cryopreservation process itself induces cell selection of “stronger” MSCs or induces greater MSC activity and expansion potential, which could translate to improved stem cell efficacy [9].

Because of the potential benefits of using cryopreserved MSCs, and the use of cryopreserved MSCs in human clinical trials, cryopreserved MSCs should be investigated in the treatment of naturally occurring tendon injury in horses. The first step in using cryopreserved MSCs in equine veterinary patients is to identify the ideal medium for cryopreservation. To do this, the effect on short-term viability and growth of MSCs post thaw must be understood [10]. Our objective was to determine whether a clinically acceptable formulation and serum source for short-term cryopreservation of equine bone marrow-derived MSCs would preserve normal viability, morphology, and normal growth kinetics post thaw. Six different freezing solutions were tested with differing serum supplementation sources and concentrations of dimethyl sulfoxide (DMSO). Different DMSO formulations were tested to determine whether a low concentration of DMSO was sufficient to preserve viability and growth of MSCs frozen in a slow-freezing method. Different serum sources were tested to determine whether an autologous serum source was sufficient to preserve viability and growth. We hypothesized that there would be no differences in the post-thaw viability, morphology, and cell growth kinetics in MSCs cryopreserved in autologous, allogeneic, or xenogeneic media or with different concentrations of DMSO.

## Methods

### Bone marrow-derived MSC isolation, expansion, and cryopreservation

All animal procedures were approved by the institution’s animal care and use committee (IACUC 2012–079). No horses were euthanized for this study. Bone marrow-derived MSCs were isolated from nine healthy horses ranging in age from 5 to 16 as described previously [11]. Briefly, bone marrow was collected from mildly sedated horses into heparinized syringes for a final concentration of 5000 units of heparin per 30 ml marrow. Red blood cell lysis was performed with ammonium chloride (7.7 mg/ml NH_4_Cl; 2.06 mg/ml hydroxymethane-aminomethane; pH 7.2) [11]. The remaining nucleated cellular portion of the marrow was plated at 175 μl original raw marrow volume/cm^2^ (Corning, Corning, NY, USA) and maintained at 37 °C, 5 % CO_2_ in humidified air. Culture medium (Dulbecco’s modified Eagle’s medium 1 g/l glucose (Mediatech, Manassas, VA, USA) supplemented with 10 % fetal bovine serum (FBS) (HyClone Inc., Logan, UT, USA), 2.5 % HEPES buffer (Corning), and 10,000 units/ml penicillin, 10,000 μg/ml streptomycin, 25 μg/ml amphotericin B (Life Technologies, Grand Island, NY, USA)) was exchanged three times per week. Once colonies or monolayers reached 70 % of confluence, cultures were passaged until there was a minimum number of MSCs available for the experiment of 3 × 10^6^ MSCs and for other experiments not outlined in this manuscript. During each passage, cells were detached from culture flasks by incubation with 5 ml per 175 cm^2^ of 0.25 % trypsin–ethylenediamine tetraacetic acid (EDTA) (Corning) for 5 minutes followed by collection, serum neutralization of trypsin with 5 ml per 175 cm^2^ of 10 % equine serum in Hank’s balanced salt solution (HBSS), and centrifugation. MSCs were resuspended in culture medium and reseeded into new tissue culture flasks at 5000 MSCs/cm^2^. Once the minimum total MSC number was achieved, MSCs were collected, rinsed by centrifugation three times, and a portion frozen in each cryopreservation solution at 10 × 10^6^ MSCs/ml. Freezing media were a formulation of 20 % serum, 10 % DMSO, and 70 % minimum essential media (MEM) (20/10/70) or a formulation of 95 % serum and 5 % DMSO (95/5). Serum sources were FBS, commercially available pooled equine serum (Thermo Scientific, Waltham, MA, USA), or autologous serum (FBS, allogenic, autologous). The same lot was used throughout the project for both FBS and pooled equine serum. After dropwise addition to the freezing solution, the cell suspension was transferred to cryovials (Thermo Scientific) which were placed immediately in a room-temperature isopropyl alcohol freeze container (Thermo Scientific), and stored in a −80 °C freezer for 24 hours. After 24 hours, the vials were transferred to liquid nitrogen storage in the liquid phase [12].

### Immunophenotyping

MSCs grown for each horse underwent immunophenotyping for expression of MHCII (Bio-Rad, Raleigh, NC, USA), CD44 (Bio-Rad), CD29 (Beckman Coulter, Brea, CA, USA), CD90 (VMRD Inc., Pullman, WA, USA), and CD45RB (VMRD Inc.) using flow cytometry. Antibodies were chosen based on previously published work and dilution of 1:400 for CD90, 1:10 for CD45RB, and 1:100 for CD44, CD29, and MHCII [13, 14]. MSCs were aliquoted at 1 million cells per Eppendorf tubes in 50 μl staining buffer.

MSCs stained with primary antibodies (MHCII, CD29, and CD44) had antibody dilution added and were incubated for 45 minutes at 4 °C. Pellets were centrifuged (400 × g, 7 brake) for 5 minutes. Pellets were washed with 200 μl Dulbecco’s phosphate-buffered saline (DPBS; Lonza, Walkersville, MD, USA) and centrifuged again before being resuspended in DPBS for analysis.

MSCs stained with secondary antibodies (CD90, CD45RB) had antibody dilution added and cells were incubated on ice for 15 minutes before being centrifuged (400 g, 4 °C) for 3 minutes. Cells were washed with 100 μl DPBS and centrifuged twice. One hundred microliters of secondary antibody dilution (1:100) was added and cells were incubated on ice in the dark for 15 minutes before being centrifuged (400 × *g*) for 5 minutes. Cells were washed with 200 μl DPBS and centrifuged again before being resuspended in 500 μl DPBS for analysis. Both primary and secondary antibodies had 5 μl 7-AAD (Biolegend, San Diego, CA, USA) added immediately prior to analysis for assessment of viability.

### Trilineage differentiation

Multipotency of MSCs used in the experiment was assessed by inducing trilineage differentiation on MSCs from eight of nine horses using techniques described previously [15–17]. For chondrogenic differentiation, three aliquots of 500,000 MSCs were centrifuged (300 × *g*, 5 minutes, 4 °C, 7 brake) in polypropylene conical tubes to form pellets. Supernatant was aspirated gently not to disturb the pellet and 1 ml chondrogenic media was added. Chondrogenic media containing Dulbecco’s modified Eagle’s medium 4.5 g/l glucose supplemented with 1 % FBS, 2.5 % hepes buffer, 10,000 units/ml penicillin, 10,000 μg/ml streptomycin, 25 μg/ml amphotericin B, 0.2 % transforming growth factor beta (Life Technologies, Grand Island, NY, USA), 301.89 μg dexamethasone (Sigma Aldrich, St. Louis, MO, USA), 50 μg/ml l-ascorbic acid (Sigma Aldrich), 40 μg/ml proline (Sigma Aldrich), and 1 % ITS premix (VWR, Radnor, PA, USA) were exchanged three times per week for 21 days. Pellets were fixed in 4 % PFA (Sigma Aldrich) for 10 minutes followed by routine embedding, sectioning, and staining with toluidine blue (Sigma Aldrich).

For adipogenic differentiation, MSCs were seeded to 10 cm plates at 1000 MSCs/cm^2^. Once the plates reached 70 % confluence, media were exchanged for adipogenic induction media for 3 days (Dulbecco’s modified Eagle’s medium F12 (VWR) supplemented with 3 % FBS, 10,000 units/ml penicillin, 10,000 μg/ml streptomycin, 25 μg/ml amphotericin B, 5 % rabbit serum (Life Technologies), 33 μM/l biotin (Sigma Aldrich), 17 μM/l pantothenate (Sigma Aldrich), 1 μM/l insulin (Sigma Aldrich), 1 μM/l dexamethasone, 225 μl isobutylmethylxanthine (Sigma Aldrich), 89 μl rosiglitazone (Sigma Aldrich)). Media were then exchanged for adipogenic maintenance media for an additional 3 days (adipogenic induction media without isobutylmethylxanthine and rosiglitazone). Induced and control plates were stained with Oil Red O (Sigma Aldrich).

For osteogenic differentiation, MSCs were seeded to 10 cm plates at 1000 MSCs/cm^2^. After reaching 70 % confluence, media were exchanged for osteogenic induction medium and maintained for 14 and 21 days (Dulbecco’s modified Eagle’s medium F12 supplemented with 10 % FBS, 10,000 units/ml penicillin, 10,000 μg/ml streptomycin, 25 μg/ml amphotericin B, 10 μM/l β-glycerophosphate (Sigma Aldrich), 20 nM/l dexamethasone, and 50 μg/ml l-ascorbic acid). Plates were stained with 2 % Alizarin Red (Sigma Aldrich).

### Thawing

After storage in liquid nitrogen for 2–5 days, vials were thawed with gentle agitation at 35 °C in a water bath until an ice ball was no longer present. Immediately post thaw, an equal volume of DPBS was added to the cell suspension. Five minutes later, the cell suspension was collected and added dropwise to 20 ml DPBS (Lonza). This thawing method was defined in a pilot project to this experiment where we determined the importance of avoiding osmotic shock in the 95/5 formulations (Additional file 1). A 100 μl aliquot of thawed cell suspension in DPBS was used for a total cell count and viability using fluorescein diacetate (67.57 mg/ml) and propidium iodide (1.35 mg/ml) in DPBS. Counting cell suspensions were plated on a Nebauer hemocytometer and visualized by fluorescence microscopy (Olympus, Center Valley, PA, USA). The live (green) and dead (red) cells were counted. A total of 10 squares were counted per sample. The cell suspension was carefully mixed by pipetting and then centrifuged to pellet the cells for removal of freezing solutions (300 × *g*, 5 minutes, 4 °C, 7 brake).

### Post-thaw cell staining with CellTrace™ label

Cell Trace™ violet dye was used to determine the speed of cellular proliferation. CellTrace™ violet dye binds to intracellular amines without interfering with cellular activity. As the cell divides, the dye is distributed equally between the two daughter cells, resulting in dye dilution that reflects the number of cell divisions which have occurred since labeling [18, 19] with CellTrace™ (Life Technologies, ThermoFisher Scientific, Grand Island, NY 14072). The amount of dye dilution in each cell and thus the number of cell divisions which have occurred, or the generation of that cell, is determined using flow cytometry. A cell that has dye dilution reflecting two cellular divisions is a second-generation cell, and so on. Post centrifugation, the supernatant was removed and pelleted MSCs were resuspended in 1 ml DPBS to be labeled with CellTrace™ violet dye, as per the manufacturer’s instructions. Briefly, cells were labeled in suspension by adding 1 μl staining solution to 1 ml DPBS containing ≤10 × 10^6^ cells. CellTrace™ violet dye and MSCs were incubated for 20 minutes at room temperature in the dark with gentle agitation every 3 minutes. Complete media were added at five times the staining volume and incubated at room temperature in the dark for an additional 5 minutes. The suspension was centrifuged, resuspended in complete media at the same volume, and incubated for another 10 minutes at room temperature in the dark, with agitation every 3 minutes. Stained MSCs were then treated as described in the following for post-thaw monolayer culture of MSCs. Life Technologies, ThermoFisher Scientific, Grand Island, NY 14072.

### Post-thaw monolayer culture of MSCs

Following labeling, MSCs from each condition were seeded at 10,000 viable MSCs/cm^2^ to tissue culture flasks (Corning) to evaluate growth kinetics, viability, and morphology and 1,000 viable MSCs were seeded to a 10 cm plate for colony-forming unit assay. Cultures were maintained as already outlined prior to cryopreservation.

Monolayer cultures of MSCs were visualized by microscopy and photographed (Olympus) using commercially available software (cellSens; Olympus). Each monolayer culture was given a morphology score of excellent (cells were spindle shaped), good (cells were wider or more star shaped), fair (cells were flattened and/or contained large vacuoles), or poor (cells were flattened, vacuolar, and foamy in appearance) and a debris score of none (<5 floating cells per 40× field), mild (<20 floating cells per 40× field floating cells), moderate (<40 floating cells per 40× field), or severe (≥40 floating cells per 40× field) by an investigator blinded to treatment group assignment (Table 1). One week after seeding the 10 cm plates, colonies were stained with 3 % crystal violet (Sigma Aldrich) and colonies were manually counted without magnification. The evaluator was masked to treatment group assignment.

**Table 1:** Age of mesenchymal stem cell donors and passage number

	Morphology	Debris
Excellent/none	Cells were spindle shaped	<5 floating cells per 40× field
Good/mild	Cells were wider or more star shaped	<20 floating cells per 40× field
Fair/moderate	Cells were flattened and/or contained large vacuoles	<40 floating cells per 40× field
Poor/severe	Cells were flattened, vacuolar, and foamy in appearance	≥40 floating cells per 40× field

### Cell generation assay

After 24 and 72 hours of monolayer culture post thaw, cells were detached and collected by addition of trypsin–EDTA. The total cell number was determined. Cells were colabeled with propidium iodide and flow cytometry was used to assess the concentration of remaining CellTrace™ cytoplasmic dye. Approximately 12,000–35,000 events were collected per condition. The cell generation with the greatest concentration of cytoplasmic dye at 24 hours post thaw was defined as the parent generation. The ModFit LT software program (Verity Software House, Topsham, ME, USA) was used to determine division rates of the MSCs. Results were reported as the current cell generation and the proportion of cells in that generation.

### Statistical analysis

Raw data were imported to a commercial statistical software program (Statistix 9; Analytical Software, Tallahassee, FL, USA). Differences between the conditions for continuous data were evaluated by one-way analysis of variance (ANOVA) with Tukey’s post-hoc tests and by Kruskal–Wallis ANOVA with pairwise comparisons as appropriate for the data structure. Differences in paired data within a group were evaluated by the Wilcoxon signed-rank test. Differences were considered significant when *p* ≤0.05.

## Results

No differences were found in the post-thaw viability, morphology, and growth kinetics of previously frozen MSCs with each of the tested solutions. Bone marrow was collected from nine mixed-breed mares aged 5–16 years. The passage number of MSCs ranged from passage 3 to passage 6 (Table 2). Differences in passage number were due to the need for different total numbers of MSCs from each horse for other experiments not outlined in this manuscript.

**Table 2:** Morphology and debris scoring system

Horse	Age	Passage
1	8	4
2	5	6
3	16	4
4	12	4
5	11	3
6	7	4
7	13	6
8	12	3
9	14	6

All assay time points were met for each donor and formulation except for Horse 6 in 95/5FBS, due to a laboratory error immediately after CellTrace™ labeling. This freezing medium (95/5FBS) was repeated later and all assays were performed. For repetition of Horse 6 95/5FBS, the same passage was available but had been cryopreserved for 18 months. Statistical significance was unchanged with the repeated data for Horse 6 in 95/5 FBS.

**Fig. 1:**
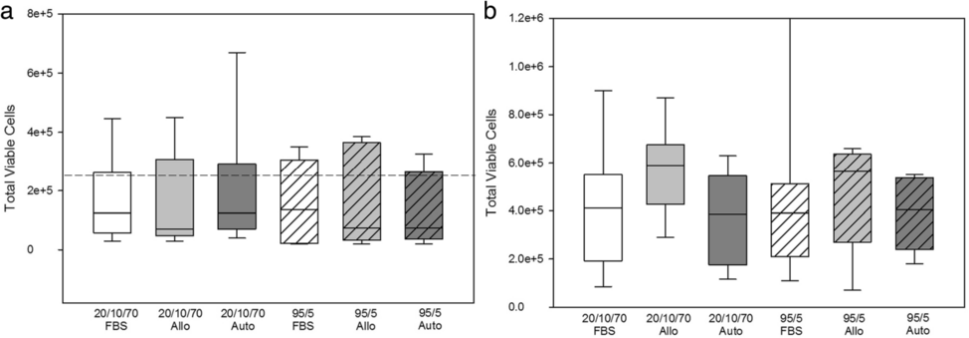
Total viable cell number. Viability of MSCs from nine horses cryopreserved in six different freezing solutions after **a** 24 hours and **b** 72 hours in monolayer culture post thaw (median, quartiles). There were no differences between the groups (24 hours, *p* = 0.96; 72 hours, *p* = 0.51). **a** *Dotted line* represents the total number of viable MSCs seeded post thaw. The total viable MSCs at 24 hours post thaw were significantly lower than the cell number seeded in all groups (20/10/70FBS, *p* = 0.02; 20/10/70Allo, *p* = 0.05; 20/10/70Auto, *p* = 0.01; 95/5FBS, *p* = 0.05; 95/5Allo, *p* = 0.05; 95/5Auto, *p* = 0.02). *Allo* allogenic, *Auto* autologous, *FBS* fetal bovine serum, *20/10/70* 20 % serum, 10 % dimethyl sulfoxide, 70 % minimum essential media, *95/5* 95 % serum, 5 % dimethyl sulfoxide

Within each solution, there were significantly fewer viable and attached MSCs at 24 hours post thaw compared with the number of viable MSCs seeded to each flask (Fig. 1; 20/10/70FBS, *p* = 0.02; 20/10/70Allo, *p* = 0.05; 20/10/70Auto, *p* = 0.012; 95/5FBS, *p* = 0.05; 95/5Allo, *p* = 0.05; 95/5Auto, *p* = 0.02).

**Fig. 2:**
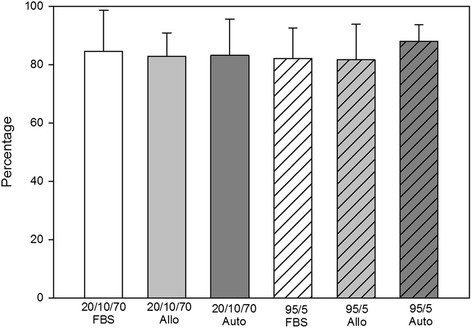
Percentage of viable cells post thaw. Viability immediately post thaw of MSCs from nine horses cryopreserved in six different solutions (mean; standard deviation). There were no significant differences between the groups. *p* = 0.844. *Allo* allogenic, *Auto* autologous, *FBS* fetal bovine serum, *20/10/70* 20 % serum, 10 % dimethyl sulfoxide, 70 % minimum essential media, *95/5* 95 % serum, 5 % dimethyl sulfoxide

**Fig. 3:**
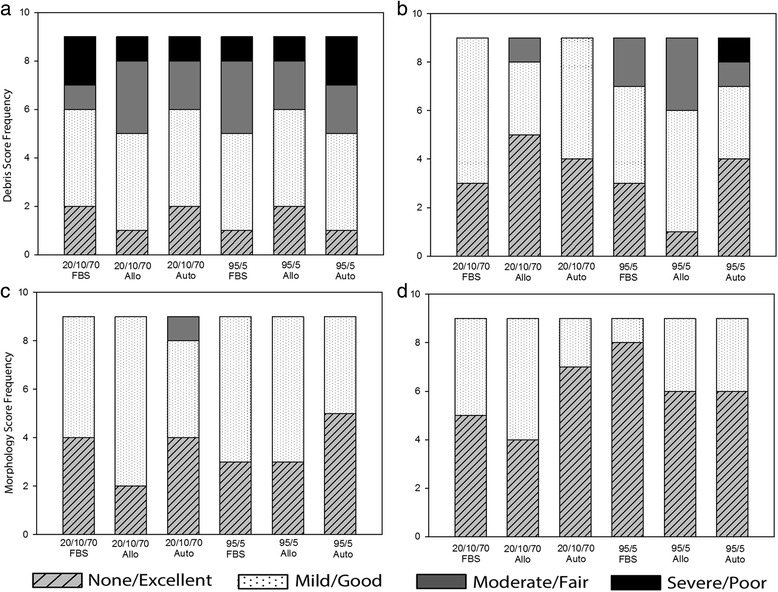
Debris and morphology scores. Frequency of **a**, **b** debris and **c**, **d** morphology scores of MSCs from nine horses cryopreserved in six different solutions in monolayer culture at **a**, **c** 24 and **b**, **d** 72 hours post thaw. *Allo* allogenic, *Auto* autologous, *FBS* fetal bovine serum, *20/10/70* 20 % serum, 10 % dimethyl sulfoxide, 70 % minimum essential media, *95/5* 95 % serum, 5 % dimethyl sulfoxide

**Fig. 4:**
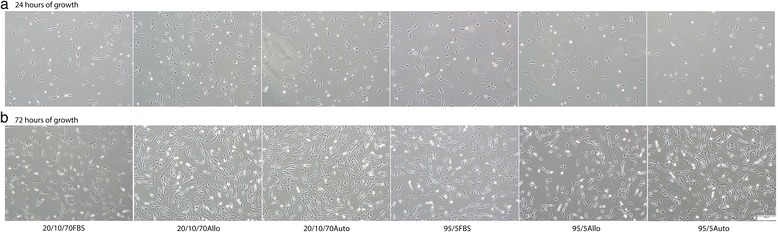
Images of monolayer culture. Microscopy images of MSCs from Horse 3 cryopreserved in six different freezing solutions after **a** 24 hours and **b** 72 hours in monolayer culture post thaw. Original magnification 40×, scale bar = 500 μm. *Allo* allogenic, *Auto* autologous, *FBS* fetal bovine serum, *20/10/70* 20 % serum, 10 % dimethyl sulfoxide, 70 % minimum essential media, *95/5* 95 % serum, 5 % dimethyl sulfoxide

**Fig. 5:**
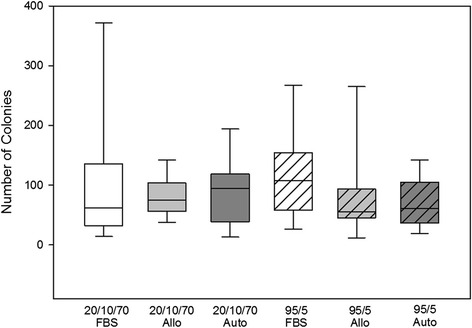
Total colony number. Numbers of colonies on CFU-F assay from MSCs cryopreserved in six different freezing solutions (median, quartiles). One thousand total viable MSCs were seeded to 10 cm plates. Colonies were stained and manually counted without magnification 1 week later. There were no differences between the groups. *p* = 0.76. *Allo* allogenic, *Auto* autologous, *FBS* fetal bovine serum, *20/10/70* 20 % serum, 10 % dimethyl sulfoxide, 70 % minimum essential media, *95/5* 95 % serum, 5 % dimethyl sulfoxide

**Fig. 6:**
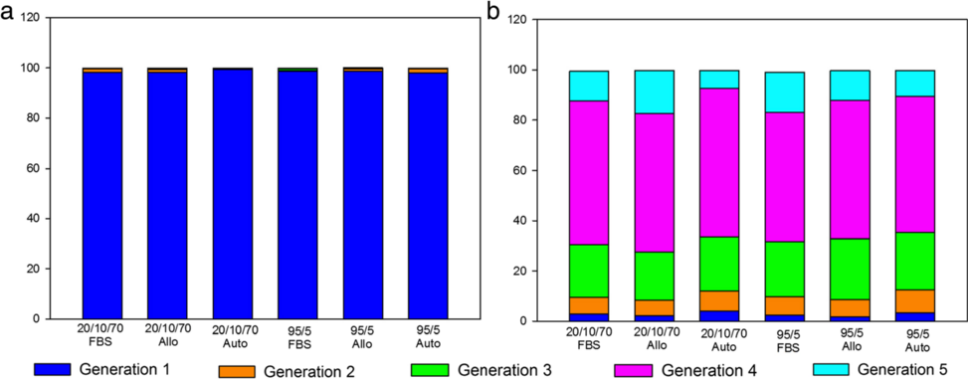
Cell generations post thaw. Percentage of MSCs cryopreserved in six different solutions in generations 1–5 at **a** 24 hours and **b** 72 hours post thaw and monolayer culture (mean). There were no differences between the groups (generation 2, *p* = 0.82; generation 3, *p* = 0.84; generation 4, *p* = 0.82; generation 5, *p* = 0.44), *Allo* allogenic, *Auto* autologous, *FBS* fetal bovine serum, *20/10/70* 20 % serum, 10 % dimethyl sulfoxide, 70 % minimum essential media, *95/5* 95 % serum, 5 % dimethyl sulfoxide

Between the solutions there were no significant differences in any of our assays. Immediate post-thaw cell viability for each condition ranged from 81 to 88 % (Fig. 2). Cell debris scores at 24 hours were generally mild to moderate debris, with one or two horses in each medium having severe debris and one or two horses in each medium with no debris (Fig. 3). MSCs receiving severe debris scores were from the same two individual donors. Horse 1 MSCs received a debris score of severe for all six freezing solutions and the total viable MSCs at 24 hours were extremely low, ranging from 25,000 to 55,000 viable MSCs. MSCs from one other individual donor (Horse 6) received severe debris scores in two formulations, 20/10/70FBS and 95/5Auto, and scores of mild and moderate for all other formulations. The cell counts at 24 hours were also very low for these formulations (145,000 and 45,000 MSCs). In contrast to scores at 24 hours, cell debris scores at 72 hours were generally none to mild (Fig. 3). Exceptions to this were the MSCs from the same two individual horses that had received severe debris scores at 24 hours. The MSCs from these horses also received worse scores at 72 hours: Horse 1 had moderate debris in all formulations except 20/10/70FBS and 20/10/70Auto, and Horse 6 had severe debris in 95/5Auto. Cell morphology scores at both 24 and 72 hours post thaw were generally good to excellent without differences between groups (Figs 3 and 4). Total viable cell counts were not different between the groups at 24 or 72 hours (Fig. 1). The percentage of confluence at 72 hours ranged from 70 to 80 % for all conditions. Numbers of colonies from the 10 cm plates ranged from 72 to 115 colonies (Fig. 5). At 24 hours post thaw, the majority (mean; standard deviation) of MSCs remained in their parent generation: 20/10/70FBS (98.4 %; 3.15), 20/10/70Allo (98.2 %; 2.36), 20/10/70Auto (99.5 %; 0.44), 95/5FBS (98.8 %; 1.48), 95/5Allo (98.6 %; 1.92), and 95/5Auto (98 %; 2.96) (Fig. 6 and Additional file 2). At 72 hours post thaw, the majority (mean; standard deviation) of MSCs were in the fourth generation: 20/10/70FBS (54.9 %; 10.25), 20/10/70Allo (55.1 %; 7.94), 20/10/70Auto (57 %; 10.32), 95/5FBS (51.5 %; 15.17), 95/5Allo (54.2 %; 12.98), and 95/5Auto (59.1 %; 11.78) (Fig. 6 and Additional file 3). When the number of MSCs contributing to the total cell number at 72 hours was calculated, based upon the mean proportion of cells in each generation at 72 hours, it was lower than the cell count at 24 hours (20/10/70FBS, 65,372; 20/10/70Allo, 95,865; 20/10/70Auto, 60,675; 95/5FBS, 80,897; 95/5Allo, 80,033; 95/5Auto, 64,578).

**Fig. 7:**
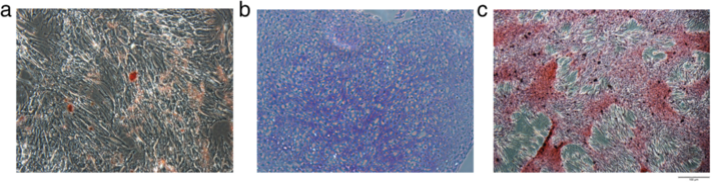
Trilineage differentiation. Images of MSCs from Horse 6 after **a** adipogenic, **b** chondrogenic, and **c** osteogenic differentiation. Trilineage differentiation was confirmed on MSCs from eight of the nine horses prior to cryopreservation. One horse did not have MSCs available to be tested. Original magnification 200×, scale bar = 100 μm

The majority of horses were negative for MHCII, CD44, and CD45RB and were positive for CD29 and CD90. Eight of nine horses underwent trilineage differentiation and were positive for osteogenic, chondrogenic, and adipogenic differentiation (Table 3; Fig. 7).

**Table 3:** Cell surface marker profiles (percent positive)

Horse	MHCII	CD44	CD29	CD45	CD90
1	1.39	68.7	100	7.27	100
2	1.41	3.19	100	1.56	99.8
3	1.52	1.88	99.8	2.5	83
4	1.13	71.8	100	5.52	89.7
5	1.24	10.8	99.4	14.9	94.7
6	1.83	9.64	100	6.42	99.5
7	60.9	18.7	100	39.5	92.8
8	5.01	48.7	99.9	21.2	90.7
9	45.4	17	99.8	17.7	99.4

## Discussion

We sought to identify whether a clinically acceptable medium for short-term cryopreservation of equine bone marrow-derived MSCs would preserve normal post-thaw viability and growth. MSCs from nine middle-aged adult horses at a broad range of passage numbers were utilized to best mimic the clinical scenario of autologous MSC therapy where differing total numbers of MSCs might be required due to differences in tendon lesion size and severity. Varying concentrations of autologous serum, pooled equine serum, or FBS; two concentrations of DMSO; and the presence or absence of a cell culture media were tested. Standard immediate and longer-term post-thaw viability assessments included total live and dead analysis, colony forming units-fibroblast (CFU-F) assay, and assessment of MSC morphology and cellular debris. A more novel analysis we used to assess growth was to stain MSC cytoplasm in a way that would not interfere with cellular activity and could be accurately measured by flow cytometry, giving us the number and frequency of cellular divisions for single cells [20]. Analysis of remaining cytoplasmic dye 24 and 72 hours after staining allowed evaluation of growth kinetics of MSCs from each cryopreservation medium, and in combination with total cell numbers and culture scoring enabled indirect assessment of post-thaw apoptosis induction.

One of the benefits of studying stem cell therapies in the horse is that the horse population, like that of man, is not homogeneous in genotype or phenotype, unlike most laboratory species. In addition to genotype and phenotype differences, individual variation in MSC characteristics, especially in species with diversity, has been reported [14, 21]. It is important to assess MSCs in models that more accurately reflect the inherent variability among human MSC preparations. Utilizing a greater number of individuals in MSC experiments better reflects responses from a diverse population. Using MSCs from nine individual donors, we found no differences between any of the freezing medium formulations in the post-thaw viability or early growth and morphology of MSCs by any of our assay methods. However, when we looked at individual horses, there were marked differences in cell expansion between the media solutions 72 hours post thaw in a few of the horses. For example, 37 % of MSCs from Horse 4 frozen in 20/10/70Allo were in generation 5, while the other five freezing solutions were much lower, ranging from 12 to 31 % of the MSC population in generation 5. As a contrasting example, only 0.5 % of MSCs from Horse 6 frozen in 20/10/70Allo had reached generation 5, while the other five freezing media had much higher percentages of MSCs in generation 5, ranging from 6 to 62 %. Had we included one of these horses in a smaller group size, we might have erroneously identified differences between the formulations.

The media formulations we tested were either 20 % serum, 10 % DMSO, and 70 % cell culture media or 95 % serum and 5 % DMSO. The 20/10/70 formulation was elected as the standard cryopreservation medium formulation used in cell culture for many cell types. Within this group, our question was whether use of xenogen-free serum sources were possible. The 95/5 formulation that has been reported recently was elected to answer two questions [22]: can an almost entirely autologous product (95 %) and a reduced DMSO concentration be used? The lack of deleterious effects when an autologous product was used with a low concentration of DMSO could move cryopreserved MSCs closer to an off-the-shelf product and would also streamline preparation of autologous MSCs.

Culture and cryopreservation of MSCs in FBS has been a standard technique for many years. Because of a desire to move toward an entirely xenogen-free product in stem cell therapies, two equine serum sources were tested. Based upon other work in our laboratory (data not shown) and that of others [7, 23], we think there are individual differences in the quality of serum for the growth of MSCs. Because of these potential variations in serum quality between individual horses, autologous serum and a commercially available pooled equine serum were tested. If individual serum quality differences exist, they do not appear to negatively affect the post-thaw viability and growth of MSCs frozen in autologous serum at either concentration we report here. Therefore, either the commercially available equine serum or autologous serum can be used for short-term xenogen-free MSC cryopreservation. An entirely autologous product versus an allogeneic, xenogen-free product would be desirable to minimize many more risks, both known and unknown.

Despite being cytotoxic and potentially toxic to the patient who will receive the cells, 10 % DMSO is the most commonly used cryoprotectant agent with or without cell washing for DMSO removal prior to cell infusion to patients [6, 24, 25]. Because of its cytotoxicity and varying reports of the effectiveness of lower DMSO concentrations in human cell cryopreservation, 5 % DMSO was also tested [26, 27]. A lower DMSO concentration, if effective, might minimize toxic effects that occur prior to freezing and in the immediate post-thaw period when MSCs are in the cryopreservation medium. Based upon our results, 5 % DMSO is sufficient as a cryoprotectant for short-term MSC cryopreservation. Using this lower concentration of DMSO would be especially important if a post-thaw rinse of MSCs was delayed or avoided altogether prior to clinical application.

Lack of differences among the cryopreservation media we tested is in stark contrast to results of a pilot project in our laboratory. In the pilot project, the same six freezing solutions and serum sources on MSCs from six middle-aged horses were tested, but we utilized a very minor variation in the thawing process. The difference in the thawing method was that post-thaw MSCs were slowly transferred in a dropwise manner to a large volume (20 ml) of DPBS, as has been reported previously, rather than the stepwise introduction to DPBS over 5 minutes we report here [8]. This minor difference in methods resulted in profound deleterious effects of cryopreservation media consisting of 95 % serum of both equine types with post-thaw viabilities of less than 60 % (Additional file 1). Susceptibility of all cell types to post-thaw osmotic shock is well known [28] and enhanced susceptibility has been suggested in human MSCs [9]. Both an absence of balanced isotonic solution and/or a lower concentration of cryoprotectant in our 95/5 formulation could have led to increased susceptibility to osmotic shock. Regardless, it appeared in our pilot project that the use of 95 % FBS was somewhat protective of the enhanced susceptibility to osmotic shock in the 95/5 formulation compared with either equine serum source. In the experiment of this report, careful handling of MSCs to reduce osmotic shock resulted in no differences among the 95/5 or 20/10/70 formulations. The importance of MSC handling immediately after the thawing process should be underscored.

**Fig. 8:**
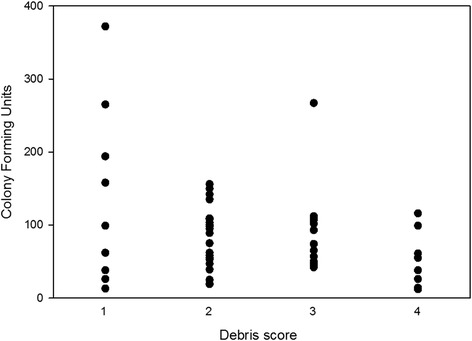
Debris score versus CFU-F. Total number of colony-forming units plotted against debris scores from MSCs cryopreserved in six different solutions and maintained in monolayer for 24 hours post thaw. When there was increased debris, there were fewer colonies 1 week later, confirming that debris consisted of MSCs which did not recover and adhere to plastic at a later time

First reported in 2011, post-thaw growth arrest of MSCs followed by a very rapid proliferation rate of surviving MSCs was seen in our study [9]. As originally suggested, this might be selection of “better” MSCs with a younger phenotype and faster proliferation rate while inducing apoptosis of the “less strong” MSCs post thaw. Our study demonstrated a lack of MSC division of the plastic adherent population in the first 24 hours with >95 % of viable MSCs still in the defined parent generation, and a greater number of nonadherent cells in the first 24 hours post thaw reflected by the higher debris scores at 24 hours and lower total cell count of adherent MSCs after 24 hours of culture than the number of MSCs seeded for all groups. These floating cells were likely apoptotic MSCs, rather than surviving but dysfunctional cells, because much higher numbers and monolayer densities would have occurred at 72 hours had the floating cells recovered function after 24 hours. Additionally, the CFU-F assay colony number was lower when debris scores at 24 hours were high (Fig. 8). This growth arrest seemed to recover between 24 and 72 hours, with the majority of viable MSCs in the fourth generation, 48 hours later. However, we think there was incomplete recovery with continued apoptosis in a portion of MSCs because the total viable cell number at 72 hours was significantly lower than one would expect given our cellular generation data. An assay of apoptosis would have been helpful to prove that apoptosis occurred. Finally, although direct comparisons with growth of MSCs from the same donors that had not been frozen were not made, our impression is that the growth during the 72 hours post thaw was much greater than we see during routine monolayer expansion of fresh MSCs. This is in contrast to a recent report where post-thaw MSC growth was not different to suspension stored MSCs where there was a steady proliferation rate for 4 days [8].

A limitation of our study was that cell surface markers, commonly used to characterize the phenotype of MSCs, and trilineage differentiation potential in vitro were not assayed post thawing. These analyses were not performed for two main reasons. First, others have reported a lack of changes in cell surface markers in fresh versus post-thaw human and porcine bone marrow-derived MSCs and that cryopreservation does not change differentiation ability [9, 29-32]. Second, others have reported lack of changes in cell surface marker profile due to serum type (autologous serum versus FBS) [33]. Therefore, we thought the minimal exposure to different media during freezing and thawing was unlikely to alter the cell surface marker expression or in vitro differentiation potential, so long as viability and growth were unchanged.

Another step that is important to note in our design is that DMSO was removed from MSCs post thaw with a post-thaw wash by centrifugation. In the clinical setting, if one used any of our tested conditions immediately post thaw, a post-thaw wash and centrifugation step would be required if removal of DMSO was desired. This washing step would require laboratory involvement in the clinical procedure, somewhat limiting the off-the-shelf availability to the treating clinician.

## Conclusion

We evaluated the short-term cryopreservation of equine bone marrow-derived MSCs in solutions consisting of differing concentrations and types of serum and differing concentrations of DMSO. A low-tech, commercially available freezing system that would be affordable in veterinary services was used. In this system, equine MSCs did not have differences in post-thaw viability and growth, regardless of the cryopreservation formulation or serum source used. The importance of minimizing osmotic shock of MSCs immediately post thaw and the potential increased risk of osmotic shock with different media for cryopreservation as found in our pilot project should be noted. Additionally, immediately post thaw there was an apparent lag phase of MSCs with little cellular division and assumed apoptosis in the first 24 hours post thaw, followed by rapid MSC growth over the next 48 hours. If a xenogen-free product with lower concentration of cryoprotectant is clinically desirable to streamline clinical and laboratory procedures by use of cryopreserved MSCs, the use of 95 % autologous serum and 5 % DMSO for the short-term cryopreservation of equine bone marrow-derived MSCs is recommended.

## Additional files

Additional file 1:
**Is a figure showing the percentage of viable cells post thaw.** Percentage of viable cells post thaw of MSCs from nine horses cryopreserved in six different solutions from our pilot project (median, quartiles). In the pilot project, there was a minor variation in the thawing process. The viable MSCs were significantly lower when MSCs were frozen in 95/5Allo and 95/5Auto solutions. (JPEG 442 kb) Additional file 2:
**Is a figure showing flow histograms of CellTrace™ dye in MSCs cryopreserved in six different freezing solutions, 24 hours post thaw and monolayer expansion.** (JPEG 285 kb) Additional file 3:
**Is a figure showing flow histograms of CellTrace™ dye in MSCs cryopreserved in six different freezing solutions, 72 hours post thaw and monolayer expansion.** (JPEG 1580 kb)

## Abbreviations

20/10/70: 20 % serum, 10 % dimethyl sulfoxide, 70 % minimum essential media
95/5: 95 % serum, 5 % dimethyl sulfoxide
ANOVA: Analysis of variance
DMSO: Dimethyl sulfoxide
DPBS: Dulbecco’s phosphate-buffered saline
EDTA: Ethylenediamine tetraacetic acid
FBS: Fetal bovine serum
HBSS: Hank’s balanced salt solution
MEM: Minimum essential media
MSC: Mesenchymal stem cell
