# Supplementary figures and images for: Cryopreservation of equine mesenchymal stem cells in 95 % autologous serum and 5 % DMSO does not alter post-thaw growth or morphology in vitro compared to fetal bovine serum or allogeneic serum at 20 or 95 % and DMSO at 10 or 5 %
Source: Stem Cell Res Ther. 2015 Nov 26;6:231. doi: 10.1186/s13287-015-0230-y (PMC4661990; doi:10.1186/s13287-015-0230-y)

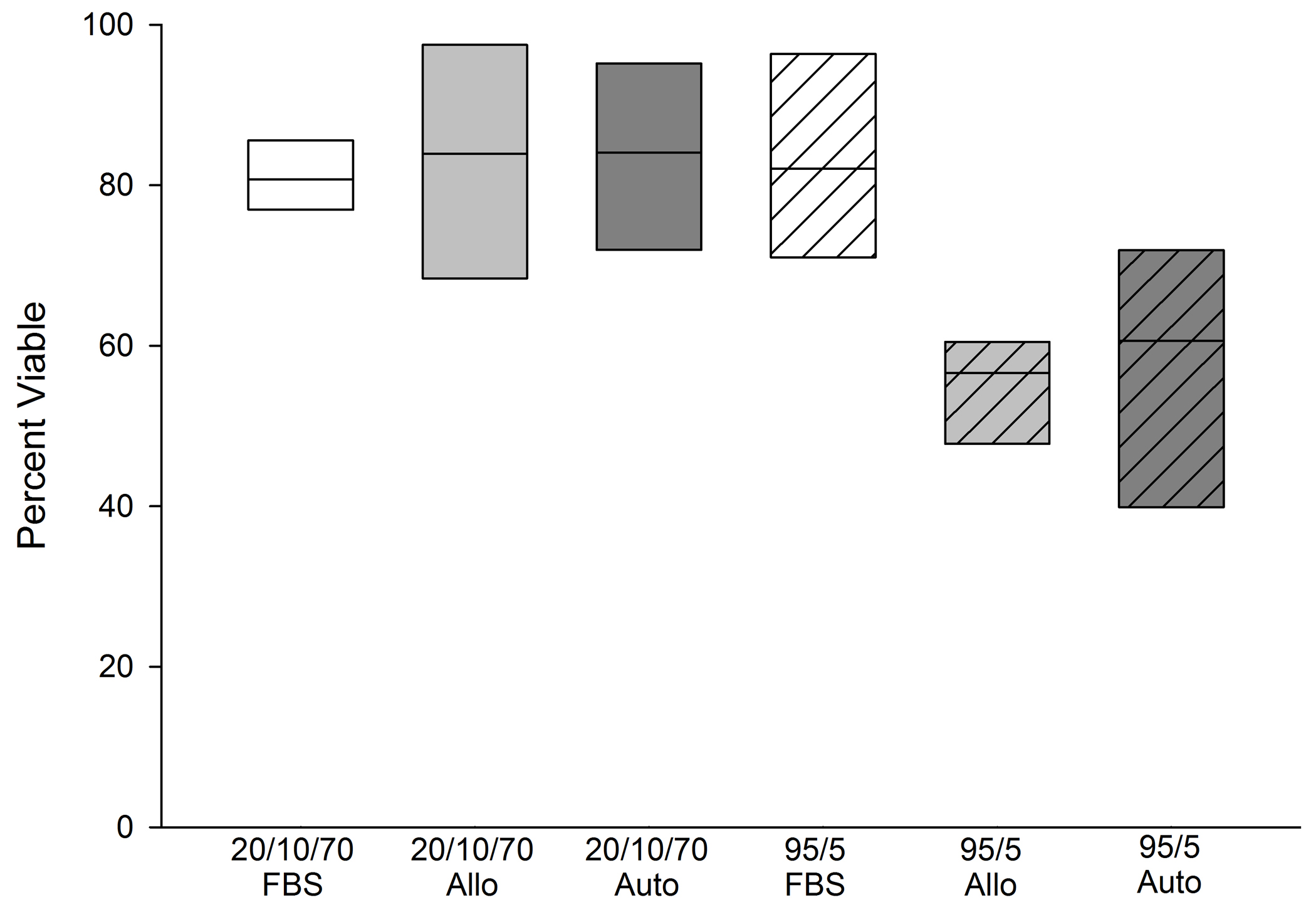

Supplement: Additional file 1: — Is a figure showing the percentage of viable cells post thaw. Percentage of viable cells post thaw of MSCs from nine horses cryopreserved in six different solutions from our pilot project (median, quartiles). In the pilot project, there was a minor variation in the thawing process. The viable MSCs were significantly lower when MSCs were frozen in 95/5Allo and 95/5Auto solutions. (JPEG 442 kb) [file 13287_2015_230_MOESM1_ESM.jpeg]

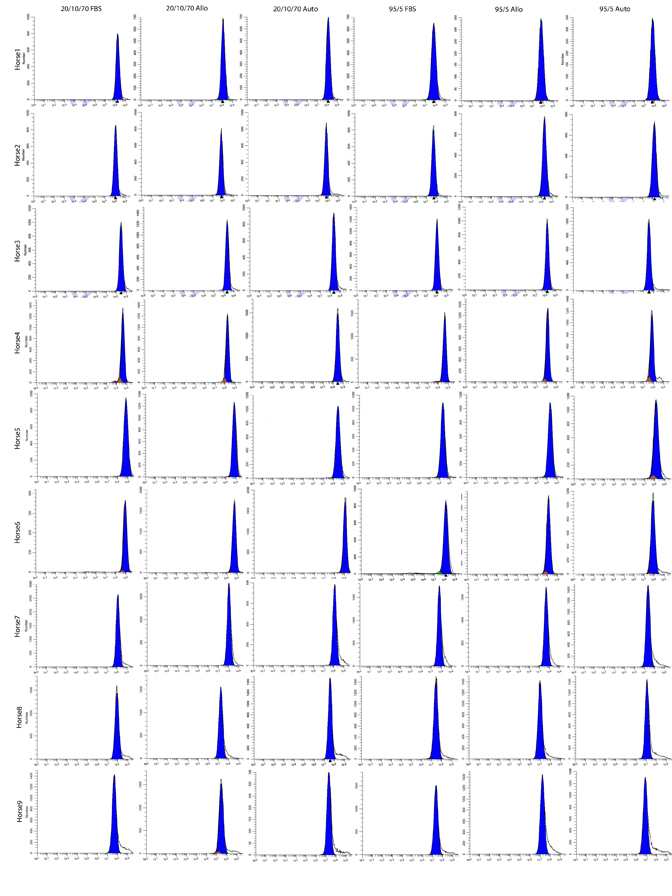

Supplement: Additional file 2: — Is a figure showing flow histograms of CellTrace™ dye in MSCs cryopreserved in six different freezing solutions, 24 hours post thaw and monolayer expansion. (JPEG 285 kb) [file 13287_2015_230_MOESM2_ESM.jpeg]

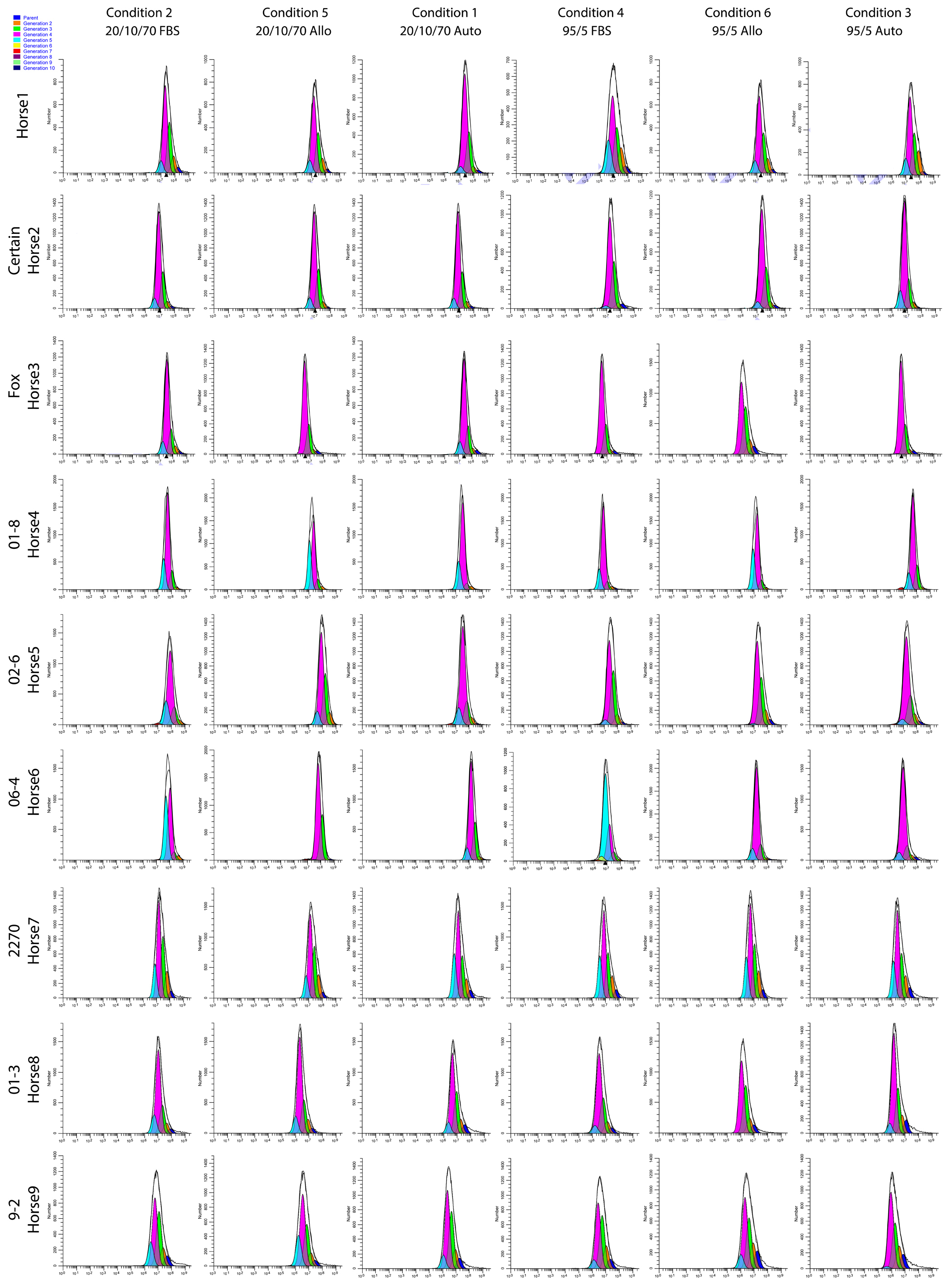

Supplement: Additional file 3: — Is a figure showing flow histograms of CellTrace™ dye in MSCs cryopreserved in six different freezing solutions, 72 hours post thaw and monolayer expansion. (JPEG 1580 kb) [file 13287_2015_230_MOESM3_ESM.jpeg]
